# Enthalpy Relaxation, Crystal Nucleation and Crystal Growth of Biobased Poly(butylene Isophthalate)

**DOI:** 10.3390/polym12010235

**Published:** 2020-01-18

**Authors:** Silvia Quattrosoldi, René Androsch, Andreas Janke, Michelina Soccio, Nadia Lotti

**Affiliations:** 1 Department of Civil, Chemical, Environmental and Materials Engineering, University of Bologna, Via Terracini 28, 40131 Bologna, Italym.soccio@unibo.it (M.S.); 2 Interdisciplinary Center for Transfer-Oriented Research in Natural Sciences (IWE TFN), Martin Luther University Halle-Wittenberg, 06099 Halle/Saale, Germany; 3 Leibniz-Institut für Polymerforschung Dresden e.V., Hohe Str. 6, 01069 Dresden, Germany; andy@ipfdd.de

**Keywords:** poly(butylene isophthalate), biopolymer, crystallization, crystal nucleation, crystal growth, enthalpy relaxation, semicrystalline morphology, fast scanning chip calorimetry (FSC), Tammann’s nuclei development method

## Abstract

The crystallization behavior of fully biobased poly(butylene isophthalate) (PBI) has been investigated using calorimetric and microscopic techniques. PBI is an extremely slow crystallizing polymer that leads, after melt-crystallization, to the formation of lamellar crystals and rather large spherulites, due to the low nuclei density. Based upon quantitative analysis of the crystal-nucleation behavior at low temperatures near the glass transition, using Tammann’s two-stage nuclei development method, a nucleation pathway for an acceleration of the crystallization process and for tailoring the semicrystalline morphology is provided. Low-temperature annealing close to the glass transition temperature (*T*_g_) leads to the formation of crystal nuclei, which grow to crystals at higher temperatures, and yield a much finer spherulitic superstructure, as obtained after direct melt-crystallization. Similarly to other slowly crystallizing polymers like poly(ethylene terephthalate) or poly(l-lactic acid), low-temperature crystal-nuclei formation at a timescale of hours/days is still too slow to allow non-spherulitic crystallization. The interplay between glass relaxation and crystal nucleation at temperatures slightly below *T*_g_ is discussed.

## 1. Introduction

Plastic production continues to grow around the world, mainly using fossil resources. The production of plastic reached 64.4 Mt in 2017 in Europe [1]. Among the various fields of the application of plastics, the packaging sector is, by far, the most important for the volumes involved. Due to the short life of plastic packaging, a huge amount of waste is accumulating in the environment, creating dramatic marine as well as terrestrial pollution problems. Decreasing finite fossil resources, and mitigating the environmental impact of plastics, are both becoming very urgent needs. In this view, bio-based materials are one of the solutions for the development of a sustainable society. In the last few years, much research had focused on the development of bioplastics, showing comparable performances in terms of cost and properties to traditional, petroleum-based plastics. Promising materials are the derivates of isophthalic acid (IPA). IPA is a bio-based building block, obtainable by the cycloaddition of bio-acrylic acid and bio-isoprene units, or directly via the fermentation of biomasses [2,3]. Solvent free polycondensation with a bio-based glycol, such as 1,4-butanediol [4,5,6], can lead to a completely bio-based homopolymer, poly(butylene isophthalate) (PBI). 

This polyester belongs to the class of poly(alkylene phthalate)s, with a chemical structure similar to the well-known isomer poly(butylene terephthalate) (PBT) [7], only differing in the position of the linkage of the phenylene group with the neighbored ester groups. While in case of PBT, the ester groups are in the *para*-position, in PBI they are in the *meta*-position.

PBI was patented in 1952 in the United States [8], and shortly afterwards, a first report about the crystallization and melting behavior of *meta*-phenylene groups containing polyesters including PBI was published [9]. With the glass transition temperature *T*_g_ being at about room temperature [10,11], any commercial use of PBI crucially depends upon its crystallization capability. Though PBI is able to crystallize [9], the maximum crystallization rate is much lower than in case of PBT. For PBT, the minimum crystallization halftimes of the order of magnitude of 0.1 and 1 s are observed at around 70 and 145 °C [12,13,14], related to crystallization via homogenous and heterogeneous crystal nucleation, respectively [14,15,16]. For PBI, in contrast, reports suggest that crystallization is fastest between around 80 and 100 °C, with the minimum crystallization halftime being of the order of magnitude of several minutes [17,18]. Regarding the structure of crystals, to date detailed information about the unit cell or conformation of chain segments are not available, despite early observation of the X-ray fiber pattern [7]. The fiber identity period was determined being 2.6 nm, pointing to a slightly distorted planar zigzag conformation of the butylene sequence with two chemical repeat units per unit cell [19]. The equilibrium melting temperature of PBI crystals is reported being between 143 °C and 165 °C, while a larger discrepancy exists regarding the bulk enthalpy of melting [9,11,17,18,20]; in a more recent study a value of 125 J/g is suggested [18]. Little is known about the morphology of PBI crystals. It may be assumed that melt-crystallization leads to formation of lamellae, since growth proceeds spherulitically; however, at an extremely low rate [19]. Further characterization of PBI concerns the segmental dynamics [21] and the rheological behavior in the molten state [22] and in solution [23,24].

Nowadays, PBI is not commercially available, though several patents report suitable industrial uses [25,26]. Research efforts often regard the derivates of PBI, such as end-capped materials, block copolymers and random copolymers [27,28,29,30,31,32,33,34], with potential application, e.g., as a hot-melt adhesive or coating. The main drawback for an industrial application of the PBI homopolymer is likely the rather low crystallization rate, which complicates obtaining semicrystalline products via melt processing, despite excellent mechanical behavior [27], good barrier properties and easy melt-processability [27]. In order to explore the possible potential of fully bio-sourced PBI for industrial uses, as well as to further understand its particularly slow crystallization with respect to its terephthalic counterpart, the present study attempts to provide a thorough analysis of the formation of semicrystalline morphologies from the melt and the glassy state. The crystallization rate and semicrystalline structure of polymers are largely dependent on the crystal nucleation. Therefore, the great part of this work is devoted to investigate the rather fast formation of crystal nuclei at high supercooling of the melt and even in the glassy state, being an alternative to the more traditional route of the acceleration of crystallization by using heterogeneous nucleators [35,36]. These nuclei then may enhance cold-crystallization, and possibly yield a non- or fine-spherulitic structure in shorter time than in the case of direct melt-crystallization [37,38]. This approach has already proven successful for other slow crystallizing polymers including poly(ethylene terephthalate) (PET) [39] or poly(l-lactic acid) (PLLA) [40,41], for which it has been shown that slow cooling allows the formation of nuclei, not yet crystals, which then grow to crystals on heating.

Fast crystal nucleation at high supercooling of the melt has been attributed to the formation of homogenous nuclei [15,16,42], however, with their detection being complicated due to their small size as well as the low enthalpy of formation. In order to overcome this problem, a defined nucleation experiment inspired by Tammann can be applied, based upon the observation that the temperatures of the maximum rate of homogenous crystal nucleation and of crystal growth often are largely different [43,44]. Tammann’s two-stage crystal nuclei development method implies the formation of nuclei at high supercooling of the melt or even in the glassy state, and their subsequent growth at higher temperature, in order to detect them. 

The method was initially applied to investigate the nucleation and crystallization of glycerol [43,44] and of organic liquids [45], and later on, of silicate glasses [46,47]. Recently Tammann´s method was proven advantageous to gain information about homogeneous nucleation in polymers, including poly (ε-caprolactone) (PCL) [48,49,50], PLLA [51,52,53], isotactic poly(butene-1) (iPB-1) [54], polyamide 6 (PA 6) [55] or PET [56]; recently, a modified version of Tammann’s approach was used to study protein crystal nucleation in solutions [57].

Homogenous crystal nucleation and even crystal growth is not restricted to temperatures above *T*_g_, but also occurs in the glassy state [47,51,54,55,58,59,60,61]. This seems of particular importance for PBI, since its melt fully vitrifies only slightly above room temperature on cooling at a rather moderate rate. Subsequent unavoidable annealing/storage of the unstable glass at ambient conditions may then allow changes of structure toward equilibrium, favored by the rather large characteristic size of cooperatively rearranging regions near *T*_g_ [61,62]. It has been found that for a large number of polymers [48,54,55,63,64,65], but also for small molecules [66], there is a well-defined sequence of enthalpy relaxation/densification of the glass [67,68], homogeneous crystal nucleation and crystal growth. A possible explanation of this observation is that the cooperative rearrangements of mobile short chain segments at the few-nanometer-length scale during enthalpy relaxation hinder the formation of crystal nuclei of supercritical size. Accordingly, in the present study, the nucleation and crystallization behavior of PBI, as well as enthalpy-relaxation experiments at temperatures slightly below *T*_g_, are included. 

The manuscript provides in the first part information about the temperature-dependence of the overall crystallization rate of PBI, measured by calorimetry, and of the crystal-growth rate, assessed by hot-stage microscopy. The second part focuses upon the analysis of crystal nucleation at temperatures near *T*_g_, employing Tammann’s analysis approach, again using calorimetry and optical microscopy. Note that the latter analysis tool, microscopy, additionally delivers valuable information about the possibility of tailoring the semicrystalline morphology of PBI by variation of the nucleation and growth pathway. This second part also includes first-time information about the morphology of PBI-crystals formed by direct melt-crystallization on one side, or by cold-crystallization, when nuclei formed at low temperature, on the other side. The third and final part of this study delivers information about the kinetics of the enthalpy relaxation of the glass, with these data allowing prediction of the onset of nuclei formation on long-term storage at ambient and sub-ambient temperatures.

## 2. Materials and Methods

### 2.1. Material Synthesis, Molecular Characterization and Processing

Dimethyl isophthalate (DMI), 1,4-butanediol (BD), and titanium tetrabutoxide (Ti(OBu)_4_) were purchased from Sigma-Aldrich, (Saint Louis, MO, USA). Poly(butylene isophthalate) (PBI) was synthesized by two-step melt polycondensation, by reacting DMI (13,200 mg, 68.1 mmol) and BD (7980 mg, 88.5 mmol). The reagents, together with Ti(OBu)_4_ (3 mg, 0.005 mmol) as a catalyst, were added in a 200 mL glass reactor, thermoset in a silicon oil bath. The reaction mixture was stirred at 100 rpm by a two-bladed centrifugal stirrer connected to an overhead motor (IKA-Werke GmbH and Co., Staufen, Germany). The temperature of the system, purged with nitrogen gas, was increased from room temperature to 200 °C, and kept constant until the 90% of the expected amount of methanol was distilled off. Then, the pressure was slowly decreased to 0.1 mbar while the temperature was increased to 220 °C. The polycondensation was considered finished when the constant torque of the stirrer was recorded for three hours. After cooling the system to room temperature, the product was purified through dissolution in chloroform, followed by precipitation in cold methanol. Finally, the as-purified polymer was dried at room temperature for 12 h.

The chemical structure of the as-synthesized polymer was confirmed by proton nuclear magnetic resonance (^1^H-NMR) spectroscopy at room temperature, employing a Varian Inova 400-MHz (Palo Alto, CA, USA). For analysis of the molar mass and polydispersity, an Agilent 1100 high-performance liquid chromatography (HPLC) System (Agilent Technologies, Santa Clara, CA, USA) equipped with a PLgel 5-μm MiniMIX-C column (Agilent Technologies, Santa Clara, CA, USA), and a refractive index detector was used. Measurements were conducted at 30 °C, eluting the system with chloroform at a rate of 0.3 mL/min. The samples were prepared by dissolving the material in chloroform, and filtering the resulting solution (2 mg/mL) through polytetra-fluoroethylene (PTFE) Millipore (0.3 µm). Polystyrene (2000–100,000 g/mol) was used to obtain a molecular-weight calibration curve. The number average molar mass and polydispersity of the used PBI were 33,150 g/mol and 2.0, respectively.

Films with a thickness of 100 µm were obtained by compression molding, using a Carver laboratory press. The polymer was sandwiched between two Teflon™ sheets and placed into the press pre-heated to 200 °C. After a dwell time of 2 min, a pressure of 5 tons/m^2^ was applied for a further 2 min, and then the sandwich was removed from the press and allowed for ballistic cooling to room temperature.

### 2.2. Instrumentation

Polarized-light optical microscopy (POM). A DMRX polarized-light optical microscope (Leica, Wetzlar, Germany) was employed to study (a) the temperature-dependence of spherulite/crystal growth rates and (b) the spherulitic superstructure/nuclei density of samples of the PBI crystallized according to Tammann’s two-stage crystal nuclei development method. The microscope was operated in transmission mode, with the samples placed between crossed polarizers.

For analysis of spherulite growth rates, a Linkam THMS 600 hot-stage (Linkam Scientific Instruments, Tadworth, UK) was used. Thin sections of 10 µm thickness were prepared from the compression-molded films using a rotary microtome (Slee, Mainz, Germany) and placed between two circular microscope coverslips of 100 μm thickness each. The glass-polymer-glass sandwich was placed into the Linkam hot-stage, and then the system was heated to 200 °C at a rate of 20 K/min. After a waiting time of 2 min, the specimen was cooled at 20 K/min to predefined isothermal crystallization temperatures between 75 and 108 °C. A single sample was used for analysis of the growth rates at different temperatures. The spherulite-growth rate was analyzed by taking images with a Motic CCD camera at discrete time intervals and plotting the spherulite diameters as a function of time.

In the case of analysis of nuclei numbers according to Tammann’s experiment, PBI specimens between the glass coverslips were quenched from 200 °C to predefined (nucleation) temperatures between 22 and 50 °C, and annealed for periods of time between 1 and 200 min. Then the system was transferred to the growth-stage temperature of 100 °C and allowed to crystallize for 20 min. Finally, after quenching to room temperature, the spherulite structure was analyzed by POM.

Fast Scanning Chip Calorimetry (FSC). FSC was used for analysis of the temperature dependencies of (a) the gross crystallization rate, (b) the nucleation kinetics at high supercooling of the melt according to Tammann’s approach, and (c) the kinetics of enthalpy relaxation of the glass. The experiments were performed using a Flash DSC 1 (Mettler-Toledo, Greifensee, Switzerland) equipped with an intracooler TC 100 (Huber, Offenburg, Germany). Specimens were prepared by cutting sections of 10 µm thickness from the 100 µm thick films using a microtome, and reduction of the lateral size of the thin section to about 50 to 100 µm with the aid of a stereomicroscope. Before placing the sample onto the membrane of the FSC chip, the sensor was conditioned and temperature-corrected according the instrument operating instructions. The sample area was purged by nitrogen gas at a flow rate of around 40 mL/min, and the sample support temperature was set to -90 °C. Further details about the instrument/sensor are available in the literature [69,70,71,72,73]. Thermal protocols/temperature–time profiles are detailed below.

Differential Scanning Calorimetry (DSC). DSC was used for analysis of the temperature-dependence of the gross crystallization rate of PBI, in order to statistically secure the data obtained by FSC. We employed a heat-flux calorimeter DSC 1 (Mettler-Toledo, Greifensee, Switzerland) connected to an Intracooler TC 100 (Huber, Offenburg, Germany). Samples with a mass of around 5 mg were punched from the compression-molded films and sealed in 20 µL aluminum pans. The furnace of the instrument was purged with nitrogen gas at a flow rate of 60 mL/min. Before starting the tests, the temperature and heat-flow-rate calibrations were checked by analysis of the melting peak of indium. PBI samples were melted by heating to 200 °C, and after equilibration of the system for 5 min, the material was cooled at 50 K/min to predefined crystallization temperatures between 70 and 105 °C, for isothermal crystallization. Data analysis included measurement of the time of maximum heat flow, that is, the peak-time of crystallization. Three independent measurements, using different samples, were performed at selected temperatures, to assure reproducibility of the data.

Atomic force microscopy (AFM). Analysis of the nanometer length-scale semicrystalline morphology of samples crystallized at 100 °C for 100 min, with the crystallization temperature directly approached by cooling the melt, or after prior annealing of the amorphous phase near *T*_g_ for 200 min, was performed with a Dimension FASTSCAN (Bruker-Nano, USA) operated in peak-force tapping mode. We employed silicon nitride sensors SCANASYST-FLUID+ (Bruker, USA) with a nominal spring constant of 0.7 N/m and a tip radius of 2 nm, using 0.02 V as set point. Specimens for AFM analysis were prepared from microtomed 10-µm-thin sections, with samples placed on a glass slide crystallized with the upper surface uncovered.

## 3. Results and Discussion

### 3.1. Spherulite and Gross Crystallization Rates of PBI

**Figure 1:**
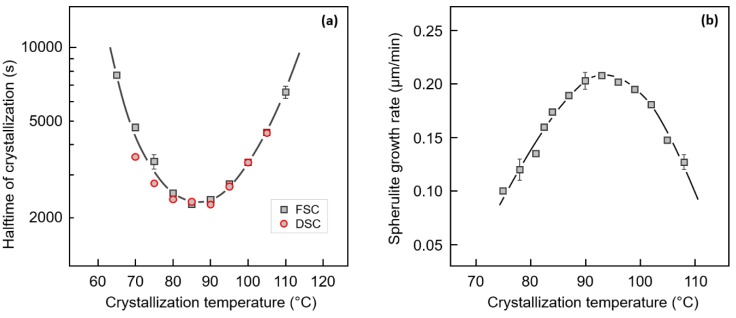
Characteristic time of crystallization (**a**) and spherulite growth rate (**b**), both as a function of the crystallization temperature. Regarding the characteristic crystallization time, fast scanning chip calorimetry (FSC) and differential scanning calorimetry (DSC) data represent the halftimes and peak-times of crystallization, respectively. In case of DSC data, the error bar is smaller than the symbol size, not shown.

Figure 1a shows characteristic times of crystallization of PBI as a function of temperature. FSC data (gray squares) were obtained by interruption of the isothermal crystallization process after predefined crystallization times, and analyzing the achieved crystallinity by the enthalpy of melting during subsequent heating. Plotting the enthalpy of melting/crystallization as a function of the crystallization time up to 10,000 s then yields the halftime of crystallization within that time-range. The plot only contains data for crystallization temperatures at which the primary crystallization process was completed. DSC data (red circles), in contrast, represent crystallization peak-times, which, however, are close to the FSC crystallization halftimes. Both data sets reveal that crystallization is fastest at 85–90 °C, with the crystallization halftime at this temperature being around 3000 s (50 min). Prior research about the temperature dependence of the crystallization rate yielded values of the temperature of the maximum crystallization rate of around 60 °C [19], 100 °C [18] and 85 °C [17], with corresponding minimum crystallization halftimes of around 12,000 s, 456 s and 210 s, respectively. The reason for the large scattering of data is unknown; it may be speculated that samples used for analysis differed in their molecular architecture, including molar mass, due to different synthesis routes, or regarding the presence of additives/stabilizers.

Figure 1b shows the spherulite growth rate of PBI as a function of temperature, as obtained by POM, revealing a maximum rate of around 200 nm/min at about 95 °C. This is the first report of the crystal growth rate of PBI, suggesting that the rather low total crystallization rate of this polyester may be caused, to large degree, by slow secondary nucleation, and not only low nucleation rate. Note that for the terephthalic, instead of the isophthalic-unit-containing polymer, PBT, the maximum spherulite growth rate is higher than 60 µm/min [74], that is, lamellar crystal growth in PBT is at least two orders of magnitude faster than in PBI.

### 3.2. Analysis of Crystal Nucleation Using Tammann’s Two-Stage Crystal Nuclei Development Method

#### 3.2.1. Optical Microscopy

**Figure 2:**
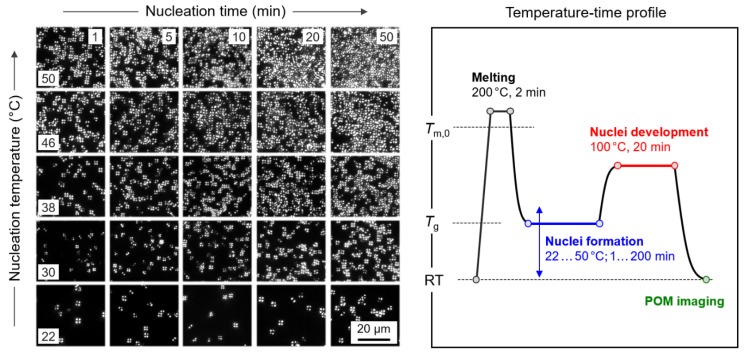
Temperature–time profile for optical analysis of isothermal nuclei formation in PBI, using Tammann’s two-stage crystal nuclei development method (right). The left part of Figure 2 shows selected polarized-light optical microscopy (POM) micrographs of PBI crystallized at 100 °C for 20 min, after nuclei formation at temperatures between 22 °C (bottom row) and 50 °C (top row) for annealing times between 1 min (left column) and 50 min (right column).

Optical microscopy was used to obtain semi-quantitative data about crystal nuclei formation in PBI using Tammann’s method. Figure 2 shows to the right schematically the temperature–time profile involving ballistic quenching of the equilibrated melt to different nucleation temperatures between 22 and 50 °C, followed by annealing up to 200 min (blue segment). After the nucleation step, the sample was ballistically heated to the development-stage temperature of 100 °C (red segment), to allow the nuclei formed in the nucleation stage, as well as any other nuclei, to growth within 20 min to countable spherulites. After rapid cooling and freezing the system, POM images were recorded. The left part of Figure 2 shows a selection of such POM images, revealing the effects of both the nucleation time (increasing from the left to the right column) and temperature (increasing from the bottom to the top row). With increasing temperature within the analyzed range from 22 to 50 °C and increasing annealing time, the number of spherulites/crystal nuclei is increasing. The observation can be interpreted such that the temperature of maximum the nucleation rate is 50 °C or higher, being just above *T*_g_ of 43 °C. The results are in general agreement with data obtained on different polymers. In fact, it was found, e.g., for PCL [48], PLLA [51,52], or PA 6 [55], that homogeneous crystal nucleation is fastest slightly above *T*_g_. Optical analysis of the nucleation behavior at high supercooling of the melt of polymers is possible only in the case of slow crystallizers, besides PBI including also PET [75] and PLLA [51]. Otherwise, the nuclei density is too high to allow spherulitic growth at the development stage, rather than leading to the formation of small nodular domains which only can be seen using higher resolution microscopic techniques [37,38,76].

#### 3.2.2. Calorimetry

**Figure 3:**
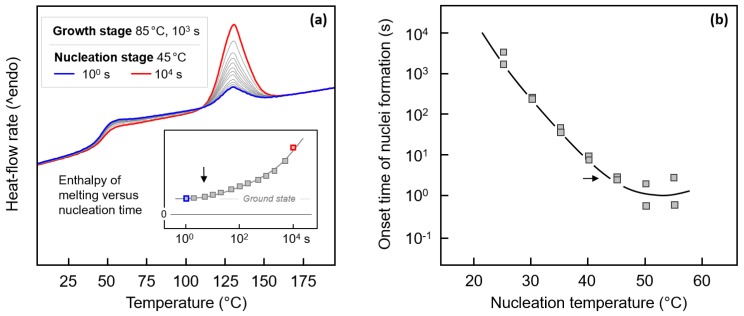
FSC heating curves, heat-flow rate as a function of temperature, of PBI, collected after subjecting PBI to Tammann’s nuclei development method (**a**). The nucleation temperature was 45 °C and the nucleation time between 1 s (blue) and 10,000 s (red). The growth-stage temperature and time were 85 °C and 1000 s, respectively, and the transfer-heating rate, that is, the rate of heating the system from 45 °C to 85 °C, was 1000 K/s. The inset shows the enthalpy of melting as a function of the nucleation time at 45 °C. The right plot (**b**) shows the onset time of nuclei formation as a function of temperature.

Quantitative data about the temperature-dependence of the nucleation kinetics were obtained by calorimetry. As an example, Figure 3 shows in the left plot a set of FSC heating curves, heat-flow rate as a function of temperature, collected after subjecting PBI to Tammann’s nuclei development method. The nucleation temperature was 45 °C and the nucleation time between 1 s (blue curve) and 10,000 s (red curve). After annealing the system at 45 °C, the material was heated at a rate of 1000 K/s to the growth-stage temperature of 85 °C, to allow the growth of nuclei to crystals for 1000 s. The amount of formed crystals is then detected on subsequent heating by the enthalpy of melting. The heating scans in Figure 3a, recorded at a rate of temperature-change of 1000 K/s, show with the step-like increase of the heat-flow rate the glass transition at around 40 °C and then endothermic melting at about 125 °C. The melting-peak area represents the fraction of crystals formed in the growth-stage of Tammann’s experiment, and is proportional to the number of nuclei present at this temperature. These include nuclei which are permanently present in the sample, nuclei formed during initial cooling of the equilibrium melt, nuclei forming in the nucleation stage, and nuclei forming during heating the system from the nucleation temperature to the growth temperature, as well as in the isothermal growth step. However, as all parameters in the various nucleation experiments are kept constant, except for the time of annealing in the nucleation stage, any change of the enthalpy of melting is attributed to the latter parameter. The inset in Figure 3a shows the change of the melting enthalpy as a function of the annealing time, with the non-zero ground state providing information about nuclei not formed during isothermal annealing; note that even in the non-annealed sample, melting is detected (blue curve). Such annealing experiments were performed at different temperatures between 25 and 55 °C. At lower temperatures than 25 °C, nucleation is too slow to be detected within the analysis-time range of 10,000 s, and at temperatures higher than 55 °C, crystal growth occurs already in the nucleation stage (see also Figure 1a). The right plot (Figure 3b) shows the onset time of nuclei formation as a function of temperature, with the horizontal arrow pointing to the nucleation experiment at 45 °C, explained with Figure 3a. The data indicate that the nucleation rate exhibits a maximum at 50–55 °C, that is, at much lower temperature than the crystal growth rate and gross crystallization rate (see Figure 1). Moreover, nucleation is fastest only slightly above the glass transition temperature, at around *T*_g_ + 25 K.

#### 3.2.3. Semicrystalline Morphology of Hot- and Cold-Crystallized PBI

**Figure 4:**
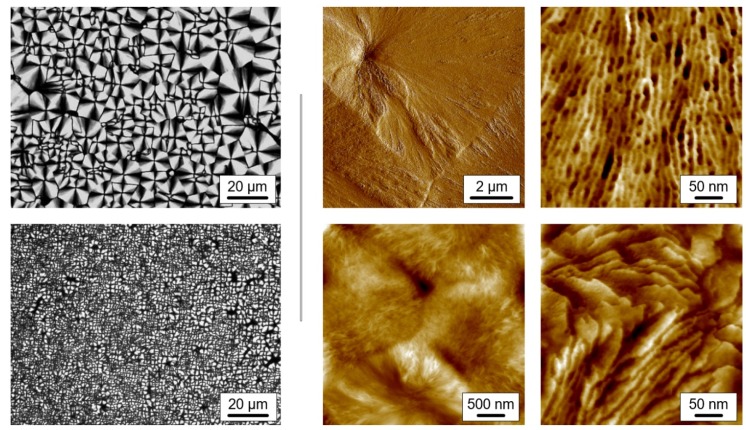
POM-images (left, b/w) and atomic force microscopy *AFM)-images (right, colored) of PBI crystallized at 100 °C. The crystallization temperature was approached either directly by cooling the melt (upper row images, hot-crystallization) or by heating the glass after annealing at 22 °C for more than 12 h, to allow nuclei formation (lower-row images, cold-crystallization).

Figure 4 illustrates the effect of the nucleation pathway on crystallization at 100 °C, demonstrating that it is an effective route to tailor the semicrystalline morphology of PBI. The left two images are POM micrographs, revealing the spherulitic superstructure obtained by direct melt-crystallization (top image, also called hot-crystallization), or by cold-crystallization (bottom image). In case of direct melt-crystallization, the equilibrium melt was quenched to 100 °C, while in the case of cold-crystallization, the material was subjected to annealing at 22 °C for more than 12 h, before crystallization at the same temperature of 100 °C. The images show clearly that cold-crystallization after prior annealing at a temperature near *T*_g_ causes the formation of a much finer superstructure than the one obtained after direct melt-crystallization. The spherulite sizes are of the order of magnitude of 10 and 2 µm after hot- and cold-crystallization, respectively, with this difference likely impacting, e.g., the mechanical properties of the material [77,78,79].

The four images to the right were obtained by AFM and show the structure at higher spatial resolution. The different size of spherulites after hot- (top images) and cold-crystallization (bottom images) is confirmed with the left two AFM-images. The upper image shows in the top left quadrant the center of a spherulite with a radius of about 6 µm, while the four spherulites in the lower image exhibit a radius of about 1 µm. In both cases, hot- and cold-crystallization, crystals are of lamellar shape, and the long dimension of the lamellae is oriented parallel to the spherulite radius. The thickness of the lamellae is of the order of magnitude of 10 nm.

### 3.3. Kinetics of Enthalpy Relaxation of the Glass of PBI

**Figure 5:**
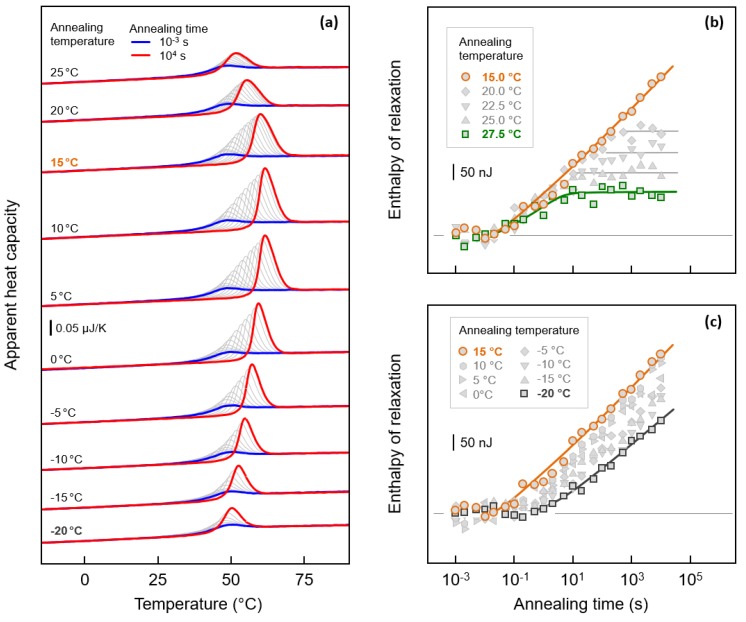
Sets of FSC heating scans, recorded at a rate of temperature-change of 1000 K/s, of PBI annealed at different temperatures between −20 °C (bottom set of curves) and 25 °C (top set of curves) for different time between 0.001 s (blue) and 10,000 s (red) (**a**). Before annealing, the sample was cooled from 180 °C, using a rate of 1000 K/s, yielding a fully amorphous sample. Enthalpy of relaxation as a function of the time of annealing at temperatures between 15 and 27.5 °C (**b**), and between −20 and 15 °C (**c**).

Figure 5a shows sets of FSC curves, recorded on heating using a rate 1000 K/s. Before heating, the relaxed PBI melt was cooled from 180 °C at 1000 K/s, which yielded fully amorphous samples. Then the obtained glasses were annealed at different temperatures between −20 °C (bottom set of curves) and 25 °C (top set of curves) for different times between 0.001 s (blue) and 10,000 s (red). Heating scans obtained on samples annealed for 0.001 s reveal with the heat-capacity step devitrification of the glass at about 42 °C. 

Only a minor enthalpy recovery-peak is detected, caused by the short residence time of the sample in the glassy state. With increasing the annealing time, the enthalpy-recovery peak, which superimposes with the heat-capacity step, increases in area due to relaxation of the glass during the prior isothermal annealing step. Quantitative information about the relaxation kinetics is obtained by evaluation of the annealing-time dependence of the area of the enthalpy-recovery peak, shown with Figure 5b,c. While Figure 5b shows data associated to annealing experiments between 15 and 27.5 °C, Figure 5c contains data of annealing experiments performed between −20 and 15 °C. Note that the relaxation-enthalpy was calculated by integrating all curves of a given curve set in a wider temperature range, covering the glass transition and the enthalpy-recovery peak, and subtracting from the obtained values the enthalpy of the sample annealed for 0.001 s.

Annealing at temperatures slightly below *T*_g_, at temperatures between 20 and 27.5 °C, allows completion of the relaxation process within 10,000 s, as indicated with the constant relaxation enthalpy after a certain annealing time, which, however, depends upon the annealing temperature (see green curve and gray horizontal lines in Figure 5b). As expected, with decreasing annealing temperature, longer time is needed to obtain a metastable structure/enthalpy-value, as well as increasing the maximum relaxation strength, that is, the maximum possible enthalpy change [67]. 

If the annealing temperature is below 20 °C, however, a stationary structural state cannot be realized within 10,000 s annealing (see orange data-points and curve). In addition, if the annealing temperature is reduced further, to temperatures lower than 15 °C (see Figure 5c), then relaxation slows down. In the case of annealing at 15 °C, relaxation begins after about 0.05 s, while on annealing at −20 °C, this relaxation only starts after about 1 s. Moreover, the slope of the curves seems to be decreasing with decreasing annealing temperature.

Important in the present study of the nucleation and crystallization behavior of PBI is the estimation of the time needed to complete the enthalpy relaxation. As described above in the Introduction, crystal nucleation in the glassy state requires that the cooperative rearrangements of the short chain segments, which occur during enthalpy relaxation, must complete, since only then can the nuclei of supercritical size form. As such, inspection of the data of Figure 5b,c leads to the conclusion that crystal nucleation in the glassy state, at temperatures of 20, 22.5, 25 and 27.5 °C, will not start on annealing shorter than about 7, 25, 100 and 500 s, respectively.

**Figure 6:**
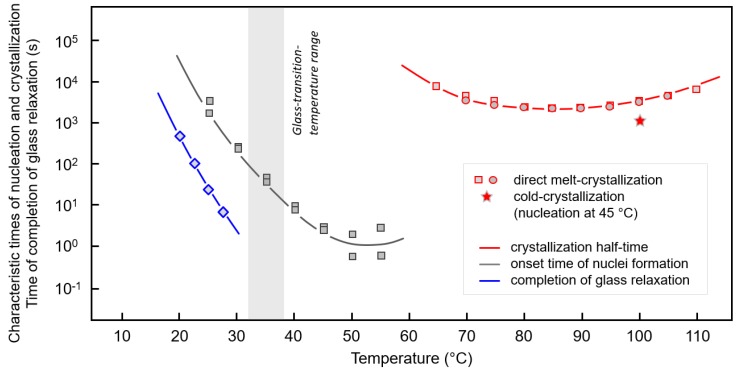
Time of completion of enthalpy relaxation (blue), onset time of nuclei formation (gray), and crystallization halftime of PBI (red) as a function of temperature. Crystallization halftimes were determined on direct melt-crystallization (see also Figure 1a, squares and circles) and after prior nuclei formation at 45 °C for different times (star symbol). The nuclei-transfer heating rate, that is, the rate of heating the nuclei from 45 °C to the growth-temperature, was 50 K/min.

As a summary, allowing an easy comparison of the kinetics of glass relaxation, crystal nucleation as well as crystallization, Figure 6 shows the time of the completion of the enthalpy relaxation (blue), onset time of nuclei formation (gray) and halftime of crystallization of PBI (red), as a function of temperature. Crystallization halftimes were determined on direct melt-crystallization (see also Figure 1a, squares and circles) and after prior nuclei formation at 45 °C for few hours (star symbol). The nuclei-transfer heating rate, that is, the rate of heating the nuclei from 45 °C to the crystallization temperature of 100 °C, was 10 K/min. In accordance with the observations described with Figure 2 and Figure 3, cold-crystallization at 100 °C, after annealing at 45 °C for less than 5 h is more than 3–5 times faster than hot-crystallization at the same temperature.

## 4. Conclusions

The crystallization behavior and the amorphous-phase dynamics of fully biobased poly(butylene isophthalate) were deeply investigated. The structural and calorimetric results confirmed its nature of a very slow crystallizing polymer due to the *meta*-configuration of the ester groups on the isophthalic moiety. The low nuclei density allows obtaining lamellar crystals and rather large spherulites through direct melt-crystallization.

Tammann’s two-stage crystal nuclei development method was successfully applied for accelerating the crystallization process via homogenous nucleation and for tailoring the semicrystalline morphology. Low-temperature annealing near the glass transition temperature leads to formation of crystal nuclei, which grow to crystals at elevated temperature and yield a much finer spherulitic superstructure than is obtained after direct melt-crystallization. As the solid-state features of a polymer deeply depend upon the amount and the kind of the ordered phases, the thermal treatments applied here offer specific ways to tailor the functional properties of the final material. The amorphous phase was also investigated, focusing on enthalpy relaxation. The study of the disordered portion evidences the densification capability of PBI at room temperature that could be responsible for the outstanding barrier properties.
